# Wavelia Microwave Breast Imaging Phase#2 Clinical Investigation: Methodological Evolutions and Multidimensional Radiomics Analysis Towards Controlled Specificity

**DOI:** 10.3390/cancers17182973

**Published:** 2025-09-11

**Authors:** Angie Fasoula, Giannis Papatrechas, Petros Arvanitis, Luc Duchesne, Julio Daniel Gil Cano, John O’Donnell, Sami Abd Elwahab, Michael Kerin

**Affiliations:** 1 Wavelia Healthcare, MVG Industries, 10563, Athens, Greece; 2 Wavelia Healthcare, MVG Industries, 91140 Villejust, France; 3 Discipline of Surgery, Lambe Institute for Translational Research, School of Medicine, University of Galway, H91TK33 Galway, Ireland; 4 Saolta University Healthcare Group, Department of Surgery, Galway University Hospital, H91YR71 Galway, Ireland

**Keywords:** microwave imaging, breast imaging, breast cancer diagnosis, medical radar, computer-aided diagnosis (CAD), image analysis, radiomic features

## Abstract

**Simple Summary:**

X-ray mammography has limited sensitivity in dense breasts. Supplemental screening and diagnostic imaging methods are required to support breast cancer diagnosis in young women, who are typically associated with high breast density, and other underdiagnosed cancer patient groups. Management of the increased rate of false positives associated with most of the supplemental breast cancer diagnosis imaging methods remains a challenge. Microwave Breast Imaging (MWBI) is an emerging imaging modality that uses non-ionizing radiation to detect and characterize breast lesions that are dielectrically contrasted against the background healthy tissue, in the microwave frequency spectrum. The Wavelia MWBI system, currently in phase#2 of development and clinical investigation, enables 3D volumetric breast imaging, semi-automated detection of breast lesions, and the generation of radar signatures associated with the histological classification of the breast lesions. It aims to improve sensitivity in young and dense breasts, combined with non-inferiority in terms of specificity, compared to 2D X-ray mammography.

**Abstract:**

Background/Objectives: The Wavelia Microwave Breast Imaging (MWBI) technology aims to increase sensitivity in dense breasts, where X-ray mammography is of limited value. Its potential contribution to the reduction in the false positives in breast cancer diagnosis, by developing MWBI image descriptors supporting malignant-to-benign lesion discrimination, is also being investigated. After a First-In-Human (FiH) study with interesting findings on a small dataset of 24 symptomatic breast lesions, an upgraded 2nd prototype of Wavelia was manufactured and tested on a larger and more diverse dataset, including 62 patients and a balanced distribution of malignant and benign symptomatic breast lesions. Methods: A set of technological and methodological evolutions, outlined in this article, was implemented in Wavelia#2 to handle the diversity in larger patient datasets. Multi-modal MWBI imaging is employed to parameterize the interaction mechanisms between the microwaves and the imaged breast at varying geometrical and tissue consistency conditions. MWBI Region-Of-Interest (ROI) extraction and characterization based on multidimensional radiomic feature vectors is implemented to expand the malignant-to-benign lesion diagnostics potential of MWBI compared to the limited scope of the FiH study with Wavelia#1, which employed three specific preselected features. Results: This study demonstrates significant diagnostic accuracy of multiple texture-based and intensity-based features to discriminate between malignant and benign breast lesions with Wavelia#2 MWBI. A phenomenological qualitative assessment of the false positive rate on healthy breasts is also presented for the MWBI technology for the first time. Conclusions: The analysis contributes to the rationalization of the MWBI imaging and image analysis outputs towards standardization, objective interpretability, and ultimate clinical acceptance.

## 1. Introduction

Breast cancer is the most common malignancy in women, affecting approximately one in eight women in their lifetime [1]. Early diagnosis is a key factor in cure and survival rates. Screening programs have drastically improved patient outcomes and prognosis through early detection and intervention, with access to mammographic screening estimated to reduce mortality rates by approximately one quarter [2].

X-ray mammography has limited sensitivity for dense breasts [3], which decreases drastically even more in cases of smaller lesions [4]. Interval cancer rates increase with breast density and are higher in women with a personal history of breast cancer [3]. The use of Digital Breast Tomosynthesis (DBT) and deep-learning (DL) computer-aided detection (CAD) software has improved outcomes on mammogram readings of dense breasts by radiologists. Nevertheless, supplemental screening and diagnostic imaging methods are required to reduce interval cancer rates and support breast cancer diagnosis in young women and high-density breasts. A small increase in interval cancer detection comes with a significant increase in recall rates for the state-of-the-art imaging modalities [5]. The management of false positives brings a substantial financial burden for the health system, as well as delays in patients’ diagnoses and treatment.

Microwave Breast Imaging (MWBI) [6,7,8,9] is an emerging imaging modality that employs low-power electromagnetic waves to scan and image the human female breast, aiming to detect, localize, and characterize breast lesions, which are dielectrically contrasted against the background healthy tissue [10,11,12,13,14,15], in the microwave frequency spectrum. The dielectric contrast, a priori increased in tissues with higher concentrations of water [16], is a physical property that has not yet been exploited in state-of-the-art diagnostic breast imaging. The diagnostic value of MWBI and its complementarity to the conventional breast imaging modalities are not fully understood [17].

As it uses non-ionizing radiation, MWBI can be particularly useful for regular and safe breast scanning at more frequent intervals compared to X-ray mammography. In addition, MWBI holds interesting potential to outperform X-ray mammography’s low sensitivity in young and dense breasts. Earlier studies of competitor MWBI systems reported an overall stable detectability rate, within the range of 70–80%, for both benign and malignant lesions, irrespective of the breast density category [18,19]. However, these early-phase promising study outcomes did not include the identification of malignancy and/or pre-malignant lesions leading to disease; the sensitivity of the MWBI modality in dense breasts needs to be better assessed.

On the other hand, the specificity of the MWBI technology remains fully unknown. The management of false positives has not been systematically addressed in any earlier MWBI study after almost three decades of active research [18,19,20,21,22,23,24,25,26,27,28,29,30,31], representing a principal barrier to the clinical validation and adoption of MWBI [20].

Going beyond state-of-the-art MWBI technologies, Wavelia is the only MWBI system that generates clinically meaningful 3D images of the breast for visual inspection by radiologists and morphological post-processing [32]. Wavelia employs multi-static radar imaging technology, enabling 3D volumetric breast imaging [33,34], semi-automated detection of breast lesions, based on intensity and persistence [32,35], and the generation of radar signatures associated with the histological classification of the breast lesions [36]. It is clearly differentiated from the active state-of-the art MWBI technologies; the two main and currently active competitor systems undergoing clinical trials [19,21,22,23,24] scan the breast on a single coronal plane to either provide a 2D representation of low spatial resolution for the full 3D breast volume being scanned or employ machine learning methods directly on the measured scattering parameters to support differentiation between malignant and benign lesions without reconstructing an image of the breast.

In its First-In-Human (FiH) clinical investigation on 24 symptomatic patients (NCT03475992) [37], Wavelia showed potential to detect and characterize breast lesions based on shape and texture descriptors of Regions-Of-Interest (ROIs) extracted from MWBI-based 3D volumetric breast images [35,36]. An upgraded prototype (Wavelia#2) [38] was subsequently manufactured and recently tested on a larger and more diverse dataset of 62 symptomatic patients, collected at the Symptomatic Breast Unit of the University Hospital of Galway, Ireland (NCT05757427) [39].

The clinical investigation results presented in this article illustrate the performance of the Wavelia#2 MWBI system in terms of discrimination between malignant and benign lesions and overall specificity. This article also focuses on the critical technological and methodological evolutions in Wavelia#2, as well as the remaining challenges, to handle the diversity of breast geometry and tissue consistency in larger patient datasets towards the standardization and clinical acceptance of the MWBI modality in the diagnostic setting. Multidimensional feature vector extraction, by plugging the PyRadiomics [40] open-source library into the ROI extraction module of the Wavelia Imaging Suite, was implemented to expand the diagnostic feature analysis compared to the initial FiH study with Wavelia#1. Malignant-to-benign lesion separability analysis using ANOVA F-statistic was conducted and demonstrated significant diagnostic accuracy for a set of texture and intensity-based features of the ROIs extracted from the Wavelia#2 MWBI 3D volumetric study images.

The ambition of the Wavelia MWBI technology is to increase the sensitivity in dense breasts, where X-ray mammography is of limited value, while contributing to a reduction in the false positive and recall rates in breast cancer diagnosis and management by developing (a) MWBI image descriptors supporting malignant-to-benign lesion discrimination and (b) a MWBI-based BIRADS score associated with malignancy risk, thus potentially expanding the classic triple assessment at breast diagnosis clinics to a novel quadruple assessment process.

## 2. Materials and Methods

### 2.1. Clinical Investigation Dataset: Diverse Study Population Profile

The Wavelia Microwave Breast Imaging (MWBI) investigational medical device was designed to scan and image the human female breast, aiming to detect, localize, and characterize breast lesions, which are dielectrically contrasted against the background healthy tissue, in the microwave frequency spectrum. In the First-In-Human (FiH) study, conducted in 24 patients [37], the Wavelia#1 prototype system demonstrated the ability to detect and discriminate between palpable breast lumps [35,36], the imaging procedure had no safety issues, and patients reported a favorable experience of the MWBI scan. The promising findings from the study, which provided the initial data to support a valid clinical association in accordance with Stage 1 of the Guidance on Clinical Evaluation (MDR)/Performance Evaluation (IVDR) of Medical Device Software (MDCG 2020-1), warranted the preparation of further clinical investigations with an upgraded prototype version of the Wavelia system (Wavelia#2) [38]. The clinical data that were collected in the 2nd study [39] with Wavelia were intended to build upon the outcomes of the FiH study and to further address the current limitations of the state-of-the-art MWBI technology applied to clinical trials at this stage.

The intended purpose of the investigational device in this pilot clinical investigation was to assess the detectability and sizing of invasive breast cancers, the detectability of benign breast lesions, and the differentiation between malignant and benign breast lesions using Wavelia#2. This study was conducted at the Symptomatic Breast Unit, University Hospital of Galway, Ireland. Female patients who presented to the symptomatic clinics with a discrete breast abnormality larger than >1 cm were assessed for participation. Patients who had surgery on either breast within the past 12 months, who had any aesthetic breast implant, or who had any active or metallic implant in their breasts were excluded from this study. Patients with very small breasts of Cup-A size and pregnant or breastfeeding patients were considered inappropriate for the imaging scanner setting and were not accepted for inclusion in this study either. The full list of patient inclusion and exclusion criteria for this study is reported in [39]. All enrolled subjects provided personal explicit written informed consent prior to any study-related procedures.

In this clinical investigation, the MWBI scan was not used for patient diagnosis and was conducted in addition to standard-of-care assessments. Standard-of-care assessments performed by the physician, as per normal practice in the symptomatic breast unit of the University Hospital of Galway, included conventional medical history, clinical assessment, breast examinations, standard-of-care reference imaging (e.g., X-ray mammogram and/or ultrasound/and or breast MRI), and core biopsy (if applicable). The written radiology reports from standard-of-care reference imaging, as well as the core biopsy reports, if applicable, were acquired and used to evaluate the performance of the MWBI system in terms of detecting and estimating the size and consistency of the dominant discrete breast abnormality of each symptomatic breast. Relevant data from Multi-Disciplinary Team (MDT) meetings in relation to the therapeutic strategy for the patient and if surgery was planned were also obtained. In the case of patients who were scheduled for surgery, key reference data from their post-surgery histology report and post-surgery MDT meeting were also collected for the evaluation of the MWBI imaging results in relation to the estimated tumor size.

A total of 73 subjects were screened. Among them, 11 patients were excluded from the final analysis: 3 due to time constraints, 2 due to device malfunction or imaging artefact from patient movement, 2 due to logistical error or discomfort in the prone position, and 4 due to breast size incompatibility with the scanner. The full analysis set comprised 62 subjects in total (84.93%). Among the 62 patients included in this study, 8 patients had bilateral radiologically reported lesions, resulting in a total of 70 lesions being analyzed for detectability and characterization by the Wavelia#2 MWBI system. In addition, 32 patients had a malignant primary lesion, 25 patients had solid benign lesions, and 5 patients presented with simple cysts.

In this exploratory study, a wide range of patient breast sizes, breast densities, and patient age profiles were included [39]. The mean age was 45.0 years (SD: 15.17) and ranged from 18 to 95 years old; 30.65% of the subjects had a breast density of D, 25.81% had a breast density of C, 22.58% had a density of B, and 3.23% had a density of A. The remaining 17.74% were of unknown breast density; no mammogram was performed as per standard-of-care assessments in the hospital due to the young age of the patient (<35 years). The mean radiological lesion size was 26.4 (mm) (SD: 10.0); 71.43% of the subjects had a biopsy clip marking the symptomatic dominant discrete lesion, and 27.42% did not. The mean breast size was 756.7 (mL) (SD: 374.43 mL).

A qualitative assessment of the breast density class, according to BIRADS Atlas 5th Edition (2013) [41], was reported by the study radiologist as part of the patient reference data. The Volumetric Breast Density (VBD) was quantitatively assessed using X-ray mammography data, where relevant as reference imaging data for this study. ‘For processing’ DICOM datafiles were processed with the Volpara Lab software package [42,43] to compute the VBD and the associated categorization of the breast per Volpara Density Grade (VDG) for the analyzed population. As part of this analysis, the volume of the breast was also computed by Volpara Lab for the subset of the study population for whom ‘For processing’ DICOM datafiles of their X-ray mammogram were made available. If mammography-based computation of the breast volume was unavailable, the Wavelia MWBI scan-based reconstruction of the breast surface [44,45] was used to compute the breast volume. A notable overestimation of the volume was reported, especially in cases of small breasts, because of no clear separation between the pendulous breast and the pectoral muscle being consistently and systematically defined at this stage of the Wavelia MWBI technology development.

This study was initiated between 16 February 2023 and 1 March 2023. First Patient First Visit (FPFV) and the first MWBI scan were completed on 9 March 2023. The final participant was scanned on 30 May 2024, and Last Patient Last Visit (LPLV) occurred on 13 June 2024. Database Lock occurred on 4 October 2024.

### 2.2. Wavelia#2 Microwave Breast Imaging (MWBI) Examination

The patient is examined lying in a prone position on the examination table of the Wavelia#2 MWBI scanner [38]. One breast is scanned at a time; both breasts of the patient are scanned sequentially for the purpose of this study. The patient’s breast under examination is immersed in a transition liquid in a cylindrical container through the circular opening of the examination table. The transition liquid is similar to a hydrating cream made of synthetic oil and water, using appropriate emulsifiers and conservatives. It is a lossy propagation medium, thus attenuating the microwaves that propagate in the exterior of the breast. The transition liquid has a dielectric constant close to that of the human skin to mitigate strong reflections on the external surface of the breast before penetrating its interior tissues for imaging.

The scanner illuminates the breast using low-power electromagnetic waves in the frequency range of 0.8–4.1 GHz and measures the fields scattered inside the breast in order to image the interior breast tissues and detect lesions based on their dielectric contrast with healthy tissues. The radiated power level by the scanner inside the cylindrical container where the breast is immersed is always lower than 60 mW. Calculations were performed to determine the localized Specific Absorption Rate (SAR) in the breast. The calculated maximum value of localized SAR, at 4 GHz for 10 g average, amounts to 0.25 W/kg and complies (safety factor 8) with the guidelines of the International Commission on Non-Ionizing Radiation Protection (ICNIRP) and the EU Council Recommendation 1999/519/EC for the limitation of exposure of the general public to electromagnetic fields. The MWBI scan is safe to be repeated as often as justified and needed to support earlier breast cancer diagnosis.

The microwave imaging scan is performed using an array of 21 wideband probes in a horizontal circular configuration. The probes are located outside the cylindrical container that hosts the transition liquid. The probe array is piloted to perform a vertical motion, such that the full vertical extent of the breast is scanned. At each 2 mm vertical scan position, each antenna sequentially illuminates the breast, while the remaining antennas sequentially receive the electromagnetic scattering at various angles around the circumference of the circular probe array in a multi-static radar imaging system configuration. The uppermost scanning position of the probe array in the Wavelia#2 prototype lies 24 mm below the horizontal level of the examination table of the scanner. In the Wavelia#2 implementation, the MWBI scan of each breast lasts between 15 and 30 min, depending on the vertical extent of the breast. This is a lengthy process; parallelization of the fully sequential probing mechanism is planned in future developments to allow clinical acceptance of such an MWBI system.

Wavelia#2 was upgraded to include the integration of an ‘aid-to-patient positioning’ module, based on a system of 6 endoscopic cameras integrated at the interior bottom of the cylindrical container of the scanner, to enable control of the positioning of the breast and ultimately enhance the quality of the MWBI scan. A software toolkit guiding the patient breast positioning in the air, before automated filling of the cylindrical container with transition liquid from the bottom, has also been developed. The toolkit is intended to verify and track the breast centering, the breast verticality, the breast azimuthal orientation, and the potentially off-centered location of the nipple on the pendulous breast to guide the Wavelia device operator through the optimization of the patient’s position for MWBI scanning.

In addition, in order to improve control over the positioning of the patient’s breast in the MWBI scanner, a short 5 to 6 min process was implemented in this clinical investigation for marking the patient’s breasts using easily applicable and removable waterproof adhesive patches. The breast marking took place before the installation of the breast in the MWBI scanner. The topology of the breast markings, defined while adopting the methodology in [46], was used to annotate useful landmark lines on the endoscopic camera data of the patient’s breast and better guide the operator through the positioning of the breast in the MWBI scanner.

Once the breast position in the scanner is fixed, images are recorded with the system of endoscopic cameras to support a valid breast quadrant definition on the MWBI images. As the MWBI images have limited spatial resolution, the data from the endoscopic cameras provide useful detail on the location of the nipple and any potential misalignment of the breast orientation to enable a more valid interpretation of the MWBI images. The optical data also provide additional information on persistent skin folds and any other identified zones of the breast, which can generate imaging artefacts during the MWBI scan. The systematic identification, rationalization, and automated management of MWBI artefacts is of significant importance for the clinical validation and acceptance of this new imaging modality. This was pointed out in earlier MWBI studies as well, which reported a very high portion—up to 33—of the MWBI scans being discarded from the analysis due to unhandled and dominating imaging artefacts [47]. The importance of the proper centering and alignment of the breast in the scanner has also been studied in detail for the breast-dedicated Magnetic Resonance Imaging (MRI) scan [48]. MRI breast imaging is performed at the prone position, and the patient setting is similar to the one applicable to Wavelia MWBI; thus, MRI ergonomic study outputs for image artefact mitigation and image quality enhancement provide useful inputs for Wavelia development.

The total duration of the Wavelia#2 MWBI examination procedure is about 1.5 h in total for both breasts, including the marking of the breasts, the positioning of the patient, the filling/emptying of the transition liquid, the microwave scan, and the cleaning of the breasts.

Significant acceleration of the full procedure is planned in future iterations of the MWBI scanner towards acceptability in the clinical setting. Acceleration of the MWBI scan itself, as earlier mentioned, combined with more efficient management of the transition liquid and automation of the breast positioning guidance procedure to minimize the required number of iterations, will be implemented. Emptying and refilling of the cylindrical container of the scanner with transition liquid between the MWBI scans of the two breasts of the patient is required in the current MWBI examination procedure, because the transition liquid is creamy and of opaque color, hindering the function of the endoscopic cameras for positioning the 2nd breast.

### 2.3. MWBI 3D Image Formation

#### 2.3.1. Sectorized Multi-Static Radar Imaging

Sectorization of the imaging scene is numerically performed in the Wavelia MWBI methodology [33,38,49] to more efficiently reconstruct the imaging scene, considering the lossy profile of the breast tissues and the transition liquid, as well as the distinct physical meaning of the forward and backward scattering mechanisms of the microwaves. A subset of 8 out of the 21 probes of the Wavelia#2 system [38] is used each time to numerically form a sub-array, the full multi-static data matrix of which is used to form a partial image of the breast per azimuthal sector of illumination. The TR-MUSIC [50] imaging algorithm is applied to form the partial image of the breast at each azimuthal sector of illumination [33]. The process is repeated 21 times; at each repetition, the center of the sub-array is numerically moved to the next adjacent probe. This way, a total of 21 partially overlapping sub-images of the breast are formed and subsequently stitched to reconstruct a coronal section of the pendulous breast, of +/2 mm thickness around the horizontal symmetry plane of the probe at the given vertical scan position. The set of partially overlapping coronal breast sections is integrated to form the full 3D image of the breast.

#### 2.3.2. Handling the Heterogeneity in the Dielectric Profile of the Breast Parenchyma

The speed of propagation of the electromagnetic waves through the breast tissues is determined by the dielectric properties (permittivity) of these tissues. The breast parenchyma is highly heterogeneous, especially in the cases of denser breasts, and its spatially varying dielectric properties are a priori unknown. A parameter (pc_fib), which has been defined as part of the Wavelia imaging methodology earlier [32,33,34,38], is directly associated with the unknown permittivity of the background medium through which the electromagnetic waves propagate in each breast imaging setting. The partial image of the breast per azimuthal sector and per coronal section of each MWBI scan is generated under varying assumptions on the parameter pc_fib. The best-fitting assumption is selected independently per imaging sector, based on image-focusing quality criteria [51].

Multiple versions of the full 3D image of the breast are generated while varying the search range for the pc_fib parameter. Expanding on the methodology that was earlier employed in the FiH clinical investigation with Wavelia#1 [32], where 5 search ranges for the pc_fib parameters were systematically employed (2 wide and 3 narrow ranges), in this clinical investigation, the 3D image of each breast is generated for a total of 11 pc_fib search ranges.

$$pc_{fib}\;ranges\;set:\{\left[10:30\right],\left[20:40\right],\left[30:50\right],\left[40:60\right],\;\left[50:70\right],\;\left[60:80\right],\;\left[70:90\right],\;\left[20:50\right],\left[50:80\right],\;\left[10:60\right],\left[40:90\right]\}\%$$

The following 3 MWBI images of each breast were systematically generated for visual inspection and interpretation by the clinical investigators and study radiologists:

- Global Averaged image: averaging all the 11 pc_fib search-range images.
- Low-permittivity image: averaging of the 4 pc_fib search-range images that involve values of $pc_{fib}\leq 50\%$.
- High-permittivity image: averaging of the 4 pc_fib search-range images that involve values of $pc_{fib}\geq 50\%$.

The concurrent review of the multiple representations serves to visually assess the persistence of a physically valid ROI while modifying the breast dielectric content assumption. The few cases in which the low-permittivity or high-permittivity image was clearly opted indicate cases of breasts lying towards either the lower or the upper border of the dielectric constant range, as considered in the Wavelia#2 MWBI software. The representation that was considered the cleanest among the three, as per visual inspection, is the one that was used as input in the Region-Of-Interest (ROI) extraction module for ROI extraction and diagnostic feature analysis.

#### 2.3.3. Wavelia MWBI 3D Imaging Output Layout

**Figure 1:**
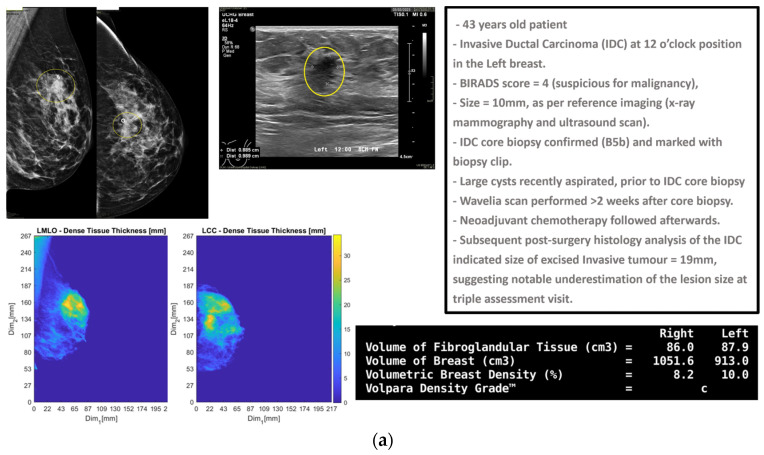

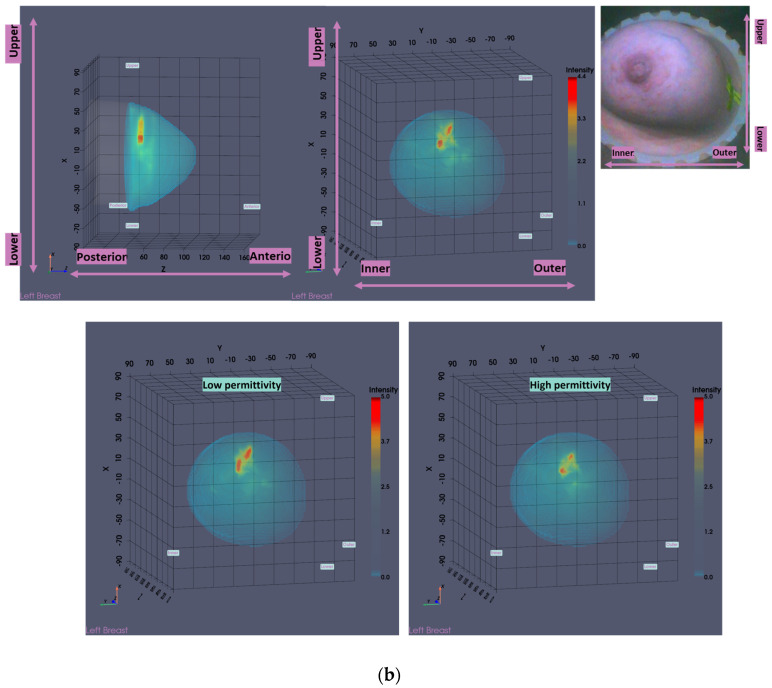
P003-L—Invasive Ductal Carcinoma: Layout of the imaging dataset: (**a**) Reference patient study data. Top row: annotated CC and MLO mammographic views of the left breast performed after the biopsy of the IDC lesion and a snapshot of the annotated ultrasound scan of the breast provided as reference data for the same lesion in this study. Bottom row: Spatial maps of the dense tissue thickness, provided as customized output of Volpara Lab on the CC and MLO mammographic planes, and Volpara Lab computational outputs to define Volumetric Breast Density (VBD). (**b**) Wavelia imaging outputs. Top row: Wavelia MWBI Global Averaged 3D volumetric image (Default View and Sagittal View) and endoscopic camera view of the breast after positioning in the MWBI scanner—the depicted view is aligned with the orientation of the MWBI 3D image’s Default View. Bottom row: Wavelia MWBI low-permittivity and high-permittivity 3D volumetric images presented in Default View.

In Figure 1, the layout of the Wavelia#2 MWBI 3D imaging results is depicted, in addition to the reference imaging and clinical data for an illustrative study case with an Invasive Ductal Carcinoma at 12 o’clock in the left breast (Patient 003, Left breast scan—P003-L).

The availability of the endoscopic camera image(s) provided as part of the Wavelia outputs was sufficient to explain the apparent misplacement of the lesion in the Wavelia MWBI images, compared to its radiological reference lesion location (as per X-ray mammography and/or ultrasound scan) in most patient cases where such ambiguity appeared. In Appendix A, one of the few study cases with an extremely misaligned position of the breast in the Wavelia MWBI scanner, resulting in difficult interpretation and explainability of the lesion’s apparent location in the Wavelia MWBI image for clinical association of the ROI with the reference, is presented. Few such cases appeared in this study, and they were all fibroadenomas (soft-tissue mobile lesions).

As part of the Wavelia MWBI scan data processing, the external envelope of the pendulous breast during the MWBI scan is first reconstructed and used to define the border of the two-propagation media: ‘in-breast’ and ‘out-of-breast’ in the MWBI imaging algorithm [44]. The quality of the MWBI-based breast contour reconstruction is highly important for the quality of the MWBI images, and it also serves to define the shape and size of the breast when inserted in the MWBI scanner. The methodological evolutions of the breast contour extraction module that were designed and implemented for the Wavelia#2 clinical investigation were recently presented in [44]. In Figure 1b, the external surface of the estimated full scanned volume is depicted in light gray color and superimposed with the Sagittal View of the 3D volumetric MWBI image while applying partial transparency.

In addition, in this clinical investigation, a specific module for automatically identifying the uppermost (i.e., the closest to the examination table) scanned breast slice, for which adequate quality of scan was assured, was employed, as an extension of the MWBI breast contour extraction module. Two main reasons for the reduced quality of the MWBI scan at the posterior section of the pendulous breast were identified during the clinical investigation and properly integrated in the module: (a) breast too large, potentially combined with sub-optimal positioning of the breast in the scanner, and (b) part of the chest wall entering the scanning zone, mainly in cases of smaller breasts. The segment that was automatically ‘cut-out’ to define the partial breast scan data for imaging, if applicable, is systematically highlighted on all the side (Sagittal) views of the Wavelia-exported images, as depicted in Figure 1b.

The low-permittivity and high-permittivity images for this breast are depicted in the bottom row of Figure 1b. While the Global Averaged image was systematically used for image analysis in this clinical investigation, the low-permittivity representation contains a cleaner and more focused Region-Of-Interest (ROI) associated with the IDC in the case of this breast.

Automated AI-driven selection among the parametric MWBI imaging outputs available in the Wavelia scan results package for more efficient handling of the heterogeneity in the breast parenchyma to further enhance the achieved breast image quality in future MWBI developments is pending.

### 2.4. MWBI Packaged Reporting per Detected Breast Lesion

#### 2.4.1. ROI Extraction and Characterization: Multidimensional Radiomics Features

The Wavelia#2 MWBI ROI extraction and validation module is based on the methodology published in [32] for breast lesion detection based on persistence, morphological image processing, and structural (volume- and solidity-based) and intensity contrast-based filtering, as introduced in the FiH clinical investigation with the Wavelia#1 MWBI prototype.

A series of operational evolutions in image post-processing [52,53] and thresholding [54] were implemented to allow more standardized feature extraction on larger datasets in the Wavelia#2 clinical investigation. In addition, going beyond [55] the limited scope of the Wavelia#1 FiH study involving the analysis of only 3 specific features, the PyRadiomics Library [40] was plugged into the Wavelia#2 ROI extraction module for a more extended multidimensional radiomic feature analysis for the ROIs extracted from the Wavelia#2 MWBI images in this clinical investigation. PyRadiomics is a comprehensive open-source library, widely used and validated by the radiomics research community. It allows the computation of a large set of first-order statistics, shape-based features (2D and 3D), and various texture features (GLCM, GLRLM, GLSZM, NGTDM, and GLDM).

**Figure 2:**
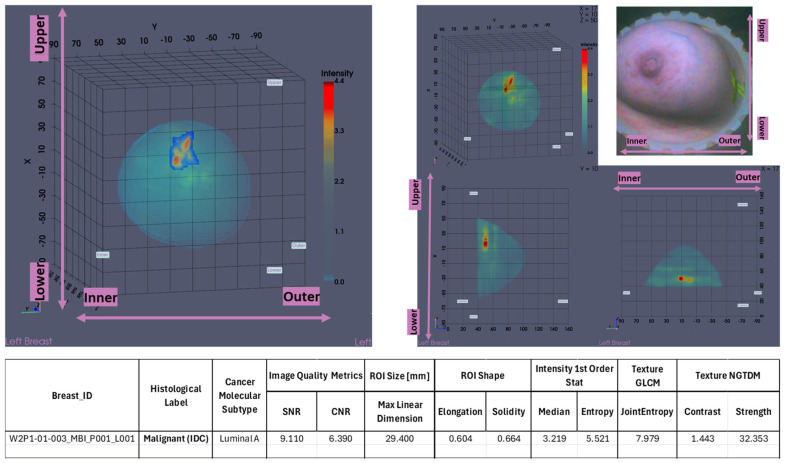
P003-L—Invasive Ductal Carcinoma: Illustrative subset of the Wavelia MWBI image analysis outputs. Packaged reporting per extracted ROI for clinical analysis. Top row: Wavelia MWBI Global Averaged 3D image’s Default View, with the extracted ROI delineated and superimposed in blue. Slices of the MWBI image on the 3 planes, cut at the centroid of the clinically relevant ROI in the MWBI image. Endoscopic camera view of the breast, aligned with the orientation of the MWBI 3D image’s Default View. Bottom Row: Tabulated ROI characteristics: columns 1–3 contain reference data, and columns 4–13 are part of the Wavelia MWBI scan analysis outputs, with data structured per extracted ROI.

The maximal linear dimension [in mm] and the volume of the ROI, as well as the Signal-to-Noise Ratio (SNR) and Contrast-to-Noise Ratio (CNR) quality metrics, were also systematically computed in the Wavelia#2 software for each extracted and validated ROI. All the information was exported as a .csv file, available as input for multidimensional statistical analysis. A table including a selected subset of the exportable features for each ROI is shown at the bottom of Figure 2.

#### 2.4.2. Wavelia MWBI 3D Image Analysis Output Layout

In Figure 2, the layout of the Wavelia MWBI 3D volumetric breast image analysis packaged output is illustrated for the same study case (P003, IDC at 12 o’clock position of the left breast).

The Wavelia MWBI scan image analysis output includes (a) the set of extracted ROIs localized with reference to the reconstructed external surface of the breast, (b) image quality metrics assessment (Signal-to-Noise Ratio (SNR) and Contrast-to-Noise Ratio (CNR)), (c) ROI size estimation: maximal linear dimension [mm], and (d) radiomic feature computation for ROI characterization: shape descriptors, 1st-order statistics of ROI intensity, Gray-Level Co-occurrence Matrix (GLCM) texture, and Neighbor Gray-Tone Difference Matrix (NGTDM) texture metrics.

### 2.5. MWBI Multi-Modal Imaging: Parameterized Interaction Mechanisms Between Microwaves and the Imaged Breast at Varying Geometrical and Tissue Consistency Conditions

A highly diverse clinical study population in terms of breast size, age, breast density, lesion size, and depth of the lesion location in the breast, as defined in Section 2.1 for the Wavelia#2 clinical investigation, is associated with a set of identified technical challenges for MWBI: (a) significantly varying distances between the probe array and the breast, (b) significantly varying levels of attenuation of the electromagnetic waves when propagating within a very dense breast or a fatty breast, and (c) significantly varying speed of the waves propagating in the highly heterogeneous breasts. Tailored data preprocessing and filtering of the MWBI scan data before feeding the imaging algorithm is required to ensure good-quality MWBI images with reliable diagnostic content in all the distinct geometrical and breast tissue consistency configurations. Three modes of operation have been defined in the context of this clinical investigation for the Wavelia#2 MWBI system.

#### 2.5.1. Wavelia#2 MWBI Scan Data Preprocessing Scheme Revisited

**Figure 3:**
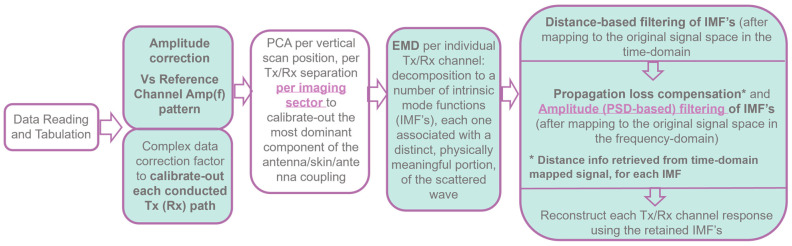
Data preprocessing and filtering scheme: Principal Component Analysis (PCA), followed by Empirical Mode Decomposition (EMD), applied to allow filtering per Intrinsic Mode Function (IMF) after mapping it either on the time- or on the frequency-domain signal space.

A block diagram with the main steps of the evolved MWBI scan data preprocessing scheme, as implemented in the Wavelia#2 scanner and outlined in [38,44], is shown in Figure 3.

Empirical Mode Decomposition (EMD) [56] for MWBI scan data preprocessing was initially introduced to the Wavelia#2 MWBI scan data processing in [38] and then again in [44], jointly focusing on the importance of the breast contour envelope for defining the border in/out of the breast for distance-based filtering. In Wavelia#2 MWBI, EMD is applied to the time-domain signal of each bistatic Tx/Rx channel computed by means of Inverse Discrete Fourier Transform (IDFT) of the measured S21 scattering parameters, as measured with a Vector Network Analyzer (VNA) in a stepped frequency sweep configuration. The time-domain signal is an analytic signal, as there are physically no negative frequencies in the spectrum of the measured frequency-domain signal. This makes it possible to apply the EMD on the real part of the time-domain signal and retrieve the complex-valued analytic signal via Hilbert transform, without any loss of information [56].

EMD is a powerful estimation tool that allows the decomposition of the signal into a number of Intrinsic Mode Functions (IMFs), each one associated with a distinct and physically meaningful portion of the scattered wave. In Wavelia#2, EMD was adopted for the first time to pre-process MWBI scan data by identifying and filtering out the residual unwanted interference of strong intensity, associated with the inter-probe coupling and/or reflections on the breast skin. These were identified as separate IMFs after mapping either in the time domain or the frequency domain of the original signal space.

- Distance-based filtering: (a) Filter out IMFs associated with residual antenna coupling: time-domain signal with maximum amplitude at very close distances to the antennas. (b) Filter out IMFs associated with radar echoes from unrealistically long distances (multipath), to reduce signal complexity and stabilize the performance of the sectorized TR-MUSIC imaging algorithm [32,33,34].
- Propagation loss compensation: Required for the Time-Reversal principle to remain practically valid. Apply the classical term, originally defined for the transmission lines. It is a multiplicative term, applied to the frequency-domain signal associated with each IMF: $e^{a\cdot d},a=\frac{2\pi f}{c_{0}}\cdot \sqrt{\frac{e_{r}}{2}\cdot \sqrt{\left(1+{tan\delta }^{2}\right)-1}}$, where *d* is the distance from which the radar echo associated with each IMF originates, *f* is the operating frequency, *c*_0_ is the speed of light, *e_r_ is* the dielectric constant, and *tanδ* is the loss tangent of the propagation medium. The propagation loss compensation term is further computed as $e^{\left(\alpha_{trans}\cdot {\hat{d}}_{OutOfBreast}+\alpha_{InBreast}\cdot {\hat{d}}_{InBreast}\right)}$, with $\alpha_{InBreast}\left(f\right)=\left(pc_{fib}\cdot \alpha_{fibroglandular}\left(f\right)+\left(1-pc_{fib}\right)\cdot \alpha_{adipose}\left(f\right)\right)$ and approximation of ${\hat{d}}_{OutOfBreast}$ and ${\hat{d}}_{InBreast}$ using the available estimate of the breast external surface and *d*, as defined in [44].
- Amplitude-based filtering: The Power Spectral Density (PSD) integrated over the full length of the signal is expected to remain constantly below a certain threshold (*PSD_tot_threh_* < 5), at nominal behavior of the MWBI system, as benchmarked during testing and validation with experimental breast phantoms [38] and then confirmed with the majority of human breast scan datasets. This fixed threshold value is not best-fitting to all the breast configurations.

#### 2.5.2. Custom Filters Defined During the Clinical Investigation

- Custom Filter#1:

The amplitude-based filtering is disabled to avoid unintentional over-filtering of the useful portion of the signal in cases of smaller breasts and/or large lesions superficially located within the breast. In these two configurations, it has been observed and experimentally confirmed that the default *PSD_tot_threh_* value may be too strict, resulting in partial elimination of radar echoes contributing to the lesion imaging and detection.

- Custom Filter#2:

As defined in [38], the default sectorization applied for multi-static radar imaging with the Wavelia#2 MWBI scanner prototype employs $N_{S}$ = 8 probes (out of the total of 21 probes covering the full circumference of the scanner) in each azimuthal imaging sector. As depicted in the block diagram in Figure 3, the Principal Component Analysis (PCA), which is performed before the EMD, employs $N_{PCA}=N_{S}+4=12$ probes by default, thus extending the imaging sector by 2 probes bilaterally.

In cases of very large breasts, if skin folds are present, generating strong artefacts in the scan, or in cases of breasts sub-optimally positioned in the scanner, thus approaching too much the probe array, a reduction in the arc length of the probe sub-array over which PCA is performed to define and subtract-out the common strong component of antenna coupling, tends to be more efficient, as more severe filtering is required. $N_{PCA}=N_{S}+2=10$ probes, thus extending the imaging sector by only 1 probe bilaterally (instead of 2 in the default setting), was applied to define the Custom Filter#2 during this clinical investigation.

**Figure 4:**
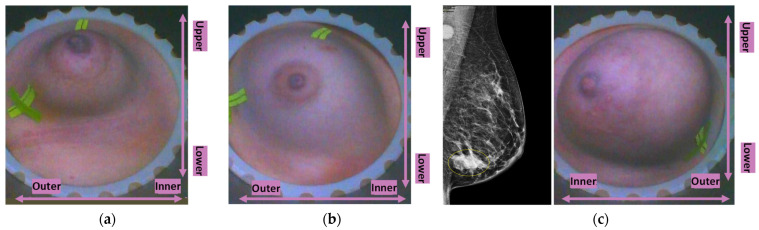
Custom Filter#1 applicability: very small breasts ((**a**) P043-R and (**b**) P011-R) and a large superficially located lesion in a medium-sized breast ((**c**) P035-L).

**Figure 5:**
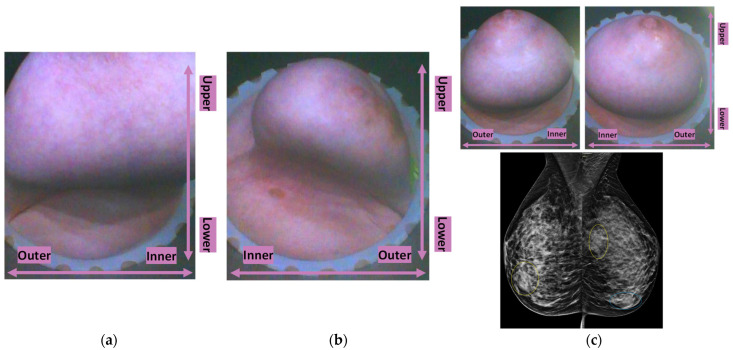
Custom Filter#2 applicability: (**a**) P039-R—very large breast, (**b**) P048-L—elder patient, breast badly positioned in the scanner with large inframammary fold being part of the scan and generating strong artefacts, and (**c**) P010—extremely dense breasts of a medium size, with bilateral cysts of low intensity being imaged.

Typical breast imaging settings in which the Custom Filter#1 or the Custom Filter#2 had the best fit are depicted in Figure 4 and Figure 5, respectively.

**Figure 6:**
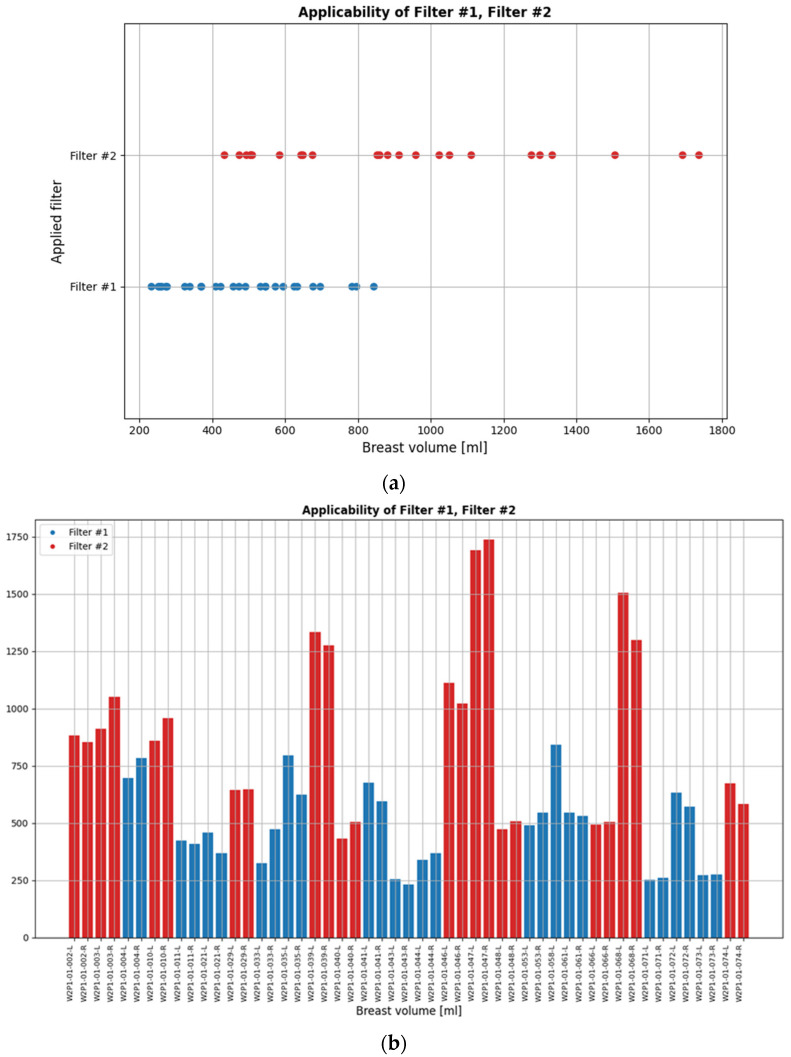
(**a**) Scanned breast volumes for which the two custom filters were selected to enhance the Wavelia MWBI scan outputs’ quality. (**b**) Patient IDs and associated breast volumes for which each of the two filters was applied.

While the default setting of the data processing scheme was applied to most patient datasets in the clinical investigation, the two custom filters were applicable to non-negligible portions of the full analysis dataset. These are indicated in Figure 6. The bilateral breast scan of the 62 evaluable patients that were included in the clinical investigation, i.e., a total of 124 breast scans, defines the full analysis dataset.

### 2.6. Malignant-to-Benign Lesion Separability Analysis

Traditional Analysis Of Variance (ANOVA) F-testing was applied for inter-class separability assessment per analyzed feature, as computed in the PyRadiomics library for each extracted ROI from the MWBI 3D breast images in this clinical investigation. ANOVA calculates the F-statistic and corresponding *p*-value. A higher F-value indicates greater variance between the groups compared to the variance within the groups, suggesting better separability. The *p*-value indicates statistical significance. This analysis quantifies how well features distinguish between two user-defined groups of labels (classes).

The focus of the analysis was on the separability assessment between the malignant and benign lesion classes for all the clinically relevant ROIs associated with the full set of malignant and benign lesions, which were detectable with the Wavelia#2 MWBI prototype in this clinical investigation. The results of this analysis are presented in Section 3.1. The indicative set of radiomic features with higher diagnostic accuracy, i.e., higher F-statistic associated with a low *p*-value, suggesting higher inter-class variability compared to the level of observed intra-class variability, thus also suggesting better separability between classes, is highlighted and discussed in the Results section (Section 3.1). An interesting tendency for separability between the two classes, mainly while employing texture features in a multidimensional space, is demonstrated with the presented results.

### 2.7. Phenomenological Qualitative Analysis of Unspecified Findings

The malignant-to-benign lesion separability analysis was further expanded in this clinical investigation data analysis to assess the separability between malignant lesions and unspecified findings (i.e., non-clinically relevant ROIs extracted from the Wavelia MWBI images).

A separate class was defined in this analysis to include all the unspecified findings.

ROI-labeling module:

A new module was developed as part of the Wavelia#2 MWBI software to be used for the labeling of all the ROIs that were extracted from the MWBI images of the bilateral breast scan of the patients. The labeled data were processed and jointly reviewed by the clinical investigators, the R&D engineering team, and an experienced radiologist/independent data reviewer.

Labeling of the ROIs was performed to be associated with either (a) the clinically relevant findings in the breast (as per the reference data), (b) scar tissue in breasts known to have been operated on in the past, or (c) understood MWBI imaging artefacts.

All non-rationalized ROIs were categorized in a separate class of ‘unspecified findings’.

MWBI imaging artefacts:

Four categories of recurrent and recognizable imaging artefacts were defined during the analysis of the Wavelia#2 MWBI study images. The corresponding labeled data were tabulated, such that they could be reported and analyzed separately. Indicative cases of bad-quality MWBI scan cases generating such imaging artefacts are depicted in Appendix B. One example for each of the four identified categories of imaging artefacts is shown.

Analysis of unspecified findings:

The rationale behind a preliminary phenomenological analysis towards the assessment of the specificity of the MWBI technology in healthy breasts is outlined below:

- First, to establish a notion of the level of outstanding/significant intensity of the ROI for each subject, the Wavelia MWBI images for the bilateral breast scan of each symptomatic patient, for whom the dominant discrete lesion in their main symptomatic breast was detectable with Wavelia#2, were simultaneously reviewed.
- The unspecified findings (i.e., non-clinically relevant ROIs) with an intensity level comparable or superior to the intensity level of the detected dominant discrete lesion ROI of each symptomatic patient were only retained for analysis.
- The unspecified findings of outstanding intensity and non-categorizable as imaging artefacts were mapped on the malignant-to-benign lesion separability feature space.
- The number of unspecified findings being confused with the malignant lesions was quantified to derive a preliminary indicator of the specificity of the new imaging modality.

## 3. Results

### 3.1. Malignant-to-Benign Lesion Separability Assessment

The Wavelia#2 clinical investigation [39] dataset, on which MWBI-based ROI characterization to assess the malignant-to-benign lesion separability was performed, included the following:

- 62 evaluable patients enrolled in this study: bilateral MWBI breast scan analyzed.
  ○ 124 MWBI breast scans in total.
- Eight patients with bilateral lesions, as per the reference clinical/radiological data.
  ○ 70 breast lesions targeted for detection and characterization with MWBI.
- 60 out of the 70 lesions detectable with MWBI.
- 59 detected lesions with a clinically relevant and validated ROI for multidimensional radiomic feature analysis and characterization with MWBI.
  ○ One study patient (P004-L) with advanced disease. Even though the MWBI imaging outputs were considered relevant by the clinical investigators, the breast zone that was affected by the disease was too extended, such that extraction of an associated and localized ROI for feature analysis and characterization was not deemed meaningful in such a case.

The full analysis set was balanced in terms of representation of malignant and benign lesions, including the following:

- 26 invasive carcinomas: 24 IDCs and 2 ILCs.
- 33 benign lesions: 26 solid benign lesions (fibroadenomas) and 7 cysts.

The separability score (ANOVA F-statistic) and its significance level (*p*-value) are reported in Table 1 for the set of features that were computed with PyRadiomics [40] and included in the malignant-to-benign lesion separability analysis.

The subset of analyzed features that showed the highest and significant diagnostic accuracy for discriminating between malignant and benign lesions in the Wavelia#2 clinical investigation dataset is highlighted in Table 1 and listed below:

First-order statistics of image intensity:
  - Entropy: It specifies the uncertainty/randomness in the image values. It measures the average amount of information required to encode the image values.
Gray-Level Co-occurrence Matrix (GLCM) texture:
  - Joint Entropy: It is a measure of the randomness/variability in neighborhood intensity values.
Neighbor Gray-Tone Difference Matrix (NGTDM) texture:
  - Strength: It is a measure of the primitives in an image. Its value is high when the primitives are easily defined and visible, i.e., an image with a slow change in intensity but larger coarse differences in gray-level intensities.

**Figure 7:**
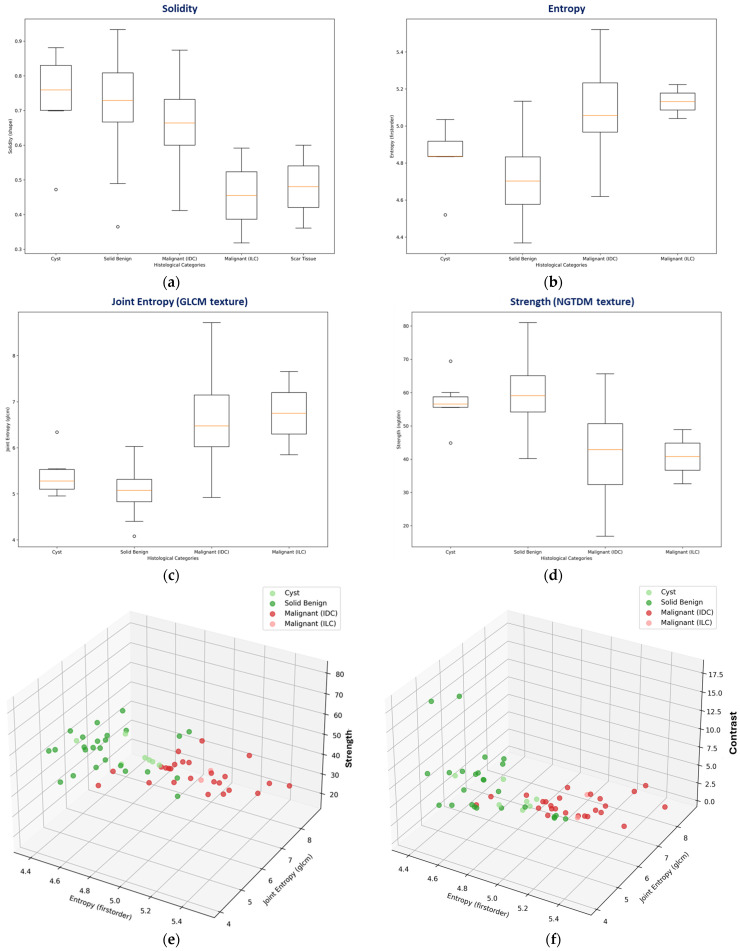
Graphical illustration of the malignant-to-benign lesion separability with Wavelia#2 MWBI: (**a**–**d**) Box-plots per analyzed feature, indicating the median, spread (IQR), range, and outlier points of each feature value distribution for 4 classes of lesions: cysts, solid benign lesions, Invasive Ductal Carcinomas (IDCs), and Invasive Lobular Carcinomas (ILCs). (**e**,**f**) Representation of the 4 classes of lesions on 3D scatter plots for two different combinations of intensity 1st-order statistics and texture features. A different color is used to represent each class on the scatter plots. The features with the highest F-statistic (separability score), as highlighted in Table 1, were selected for the graphical illustration. Higher-dimensional analysis is possible.

Box-plots representing the distributions of the most discriminative features per lesion class (i.e., histological type) are shown in Figure 7b–d. A box-plot for the solidity shape descriptor, which was employed during the Wavelia#1 FiH study as the main discriminator between malignant and benign lesions [36], is also shown in Figure 7a. Cysts show a clear tendency for higher solidity values, while ILCs and scar tissue (i.e., the non-mass-like ROIs) show a tendency for separability in terms of lower solidity. However, limited diagnostic accuracy for the discrimination between IDCs and solid benign lesions is reported while analyzing the solidity feature on the larger and more diverse dataset of the 2nd clinical investigation. Three-dimensional scatter plots, allowing for exploring the relationship between three features simultaneously, are depicted in Figure 7e,f for two different combinations of features.

### 3.2. Typical Malignant and Benign Lesions: MWBI Images and Extracted Lesion Features

In this section, the characteristics of four typical cases of breast lesions (two malignant and two benign) with good-quality MWBI scans, resulting in a clear representation of these lesions as malignant or benign in the Wavelia feature analysis space, are discussed.

**Figure 8:**
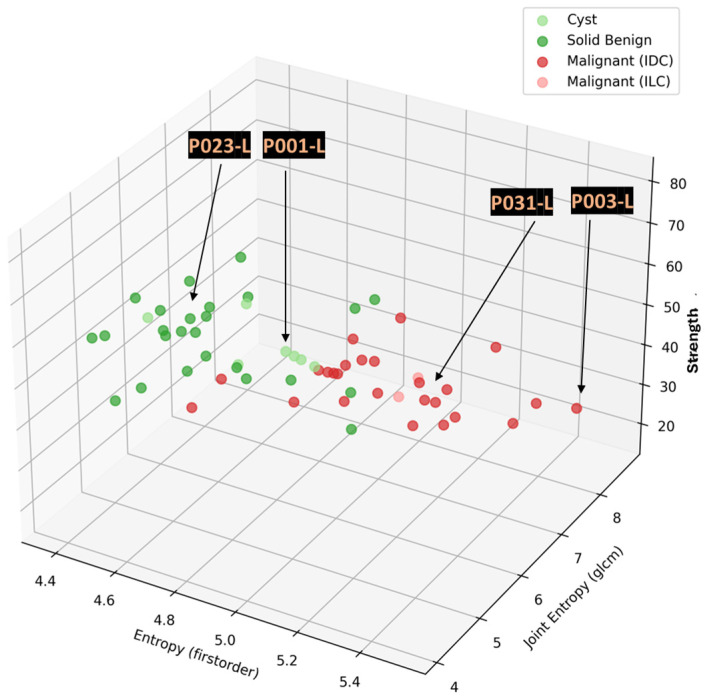
Highlighting the 4 lesions on the Wavelia MWBI feature analysis space: an indicative 3D feature space depicted, considering three of the features with the highest diagnostic accuracy in terms of discriminating malignant against benign lesions.

The principal characteristics of these lesions and a representative subset of the features that were computed for the associated ROIs, as extracted from the MWBI images, are summarized in Table 2. The points that represent the four lesions on the scatter plot of an indicative three-dimensional representation of the Wavelia MWBI feature analysis space are highlighted and labeled in Figure 8.

**Figure 9:**
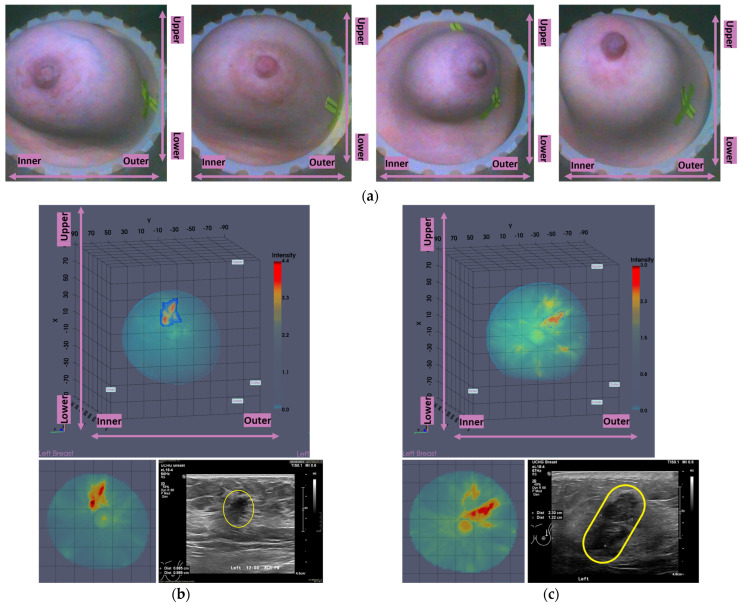

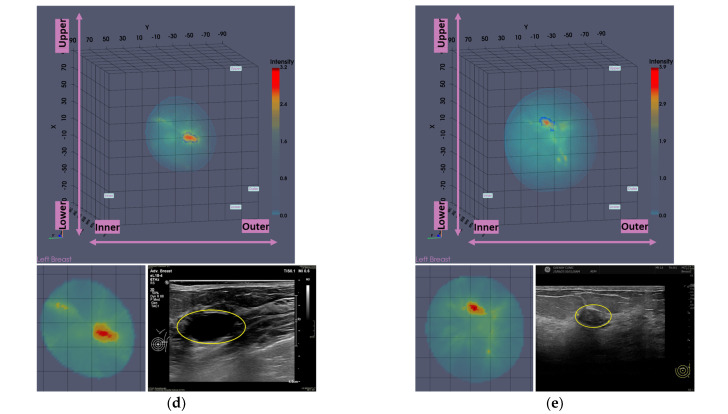
Reference and Wavelia MWBI images for the 4 typical cases of good-quality Wavelia MWBI images with clear characterization as malignant or benign lesions in the Wavelia feature analysis space: (**a**) Endoscopic camera views of the 4 breasts after positioning in the MWBI scanner—depicted camera views are aligned with the orientation of the MWBI 3D image’s Default View. (**b**) P003-L IDC—Luminal A. (**c**) P031-L IDC—Luminal B. (**d**) P001-L—Cyst. (**e**) P023-L—Fibroadenoma. (**b**–**e**): Wavelia MWBI 3D image’s Default View, with the extracted ROI delineated and superimposed in blue, coronal XY slice of the MWBI image cut at the centroid of the clinically relevant ROI in the MWBI image, and snapshot of the ultrasound scan of the breast provided as reference data for the same lesion in this study.

An illustrative subset of the Wavelia MWBI imaging outputs, together with the ultrasound reference scan, is depicted in Figure 9 for each of the four cases. In Figure 9a, endoscopic camera views showing the position and orientation of the four breasts in the MWBI scanner are depicted.

A quite stable Signal-to-Noise Ratio (SNR) level, in conjunction with overall comparable Contrast-to-Noise Ratio (CNR) levels, slightly increased in the case of Patient 003 (IDC, molecular subtype Luminal A), is observed in Table 2 for the four cases. The SNR measures the ratio of the mean signal intensity within the ROI to the standard deviation of the background noise. A higher SNR generally indicates a better image quality. The CNR measures the difference between the mean ROI intensity and the mean background intensity, relative to the background noise. CNR quantifies how well the ROI stands out from its surroundings. An insightful comparison of these levels to the ones of the images in confusing cases of lesions, which are not clearly identifiable as malignant or benign on the Wavelia MWBI feature analysis space, is provided in the following section.

Good correspondence between the morphology and maximal linear dimension of the extracted ROIs from the Wavelia MWBI volumetric images and the ultrasound reference representations of the lesions is notable by comparative visual inspection of the images in Figure 9c–e. The case in Figure 9b is the same case used in Section 2 to illustrate the layout of the Wavelia MWBI imaging and image analysis outputs in Figure 1 and Figure 2, respectively. Based on the more detailed clinical reference information provided for this case in Figure 1a, there is clear evidence that the ultrasound scan provided only partial visibility of this IDC, resulting in a severe underestimation of its size in this case. The 19 mm size of the excised tumor, as per the post-surgery histology report, also considering that the patient underwent neoadjuvant chemotherapy before surgery and after the Wavelia MWBI scan of the breast, renders the MWBI-based estimate of the lesion size relevant and worth highlighting in this case.

### 3.3. Confusing Cases Due to High Breast Density and/or Challenging Lesion Location in the Breast

**Figure 10:**
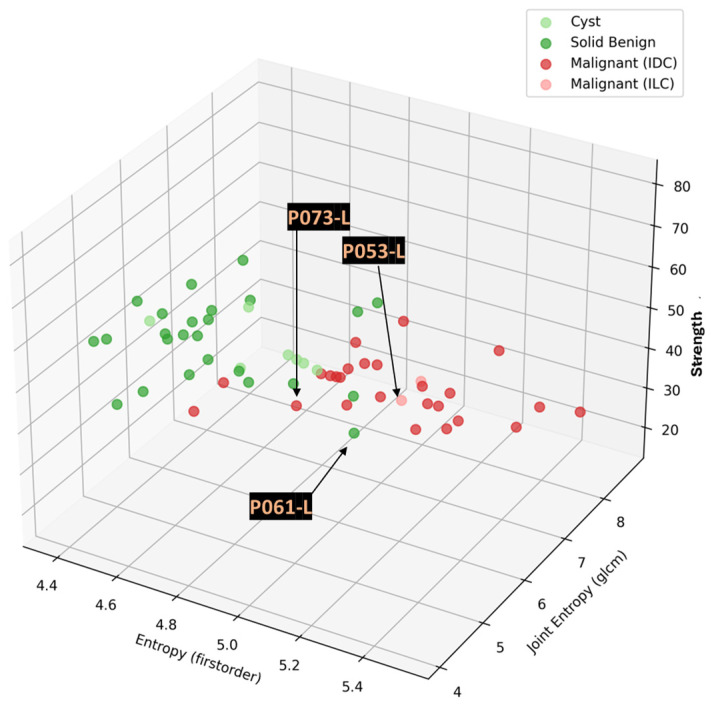
Highlighting the 3 lesions on the Wavelia MWBI feature analysis space: an indicative 3D feature space depicted, considering three of the features with the highest diagnostic accuracy in terms of discriminating malignant against benign lesions.

In this section, the characteristics of three unclear cases of breast lesions (two malignant and one benign) with moderate-quality MWBI scans, resulting in a confusing representation of these lesions as malignant or benign in the Wavelia feature analysis space, are presented. The principal characteristics of these lesions and a representative subset of the features that were computed for the associated ROIs, as extracted from the MWBI images, are summarized in Table 3. The points that represent the three lesions on the scatter plot of the Wavelia MWBI feature analysis space are highlighted and labeled in Figure 10.

**Figure 11:**
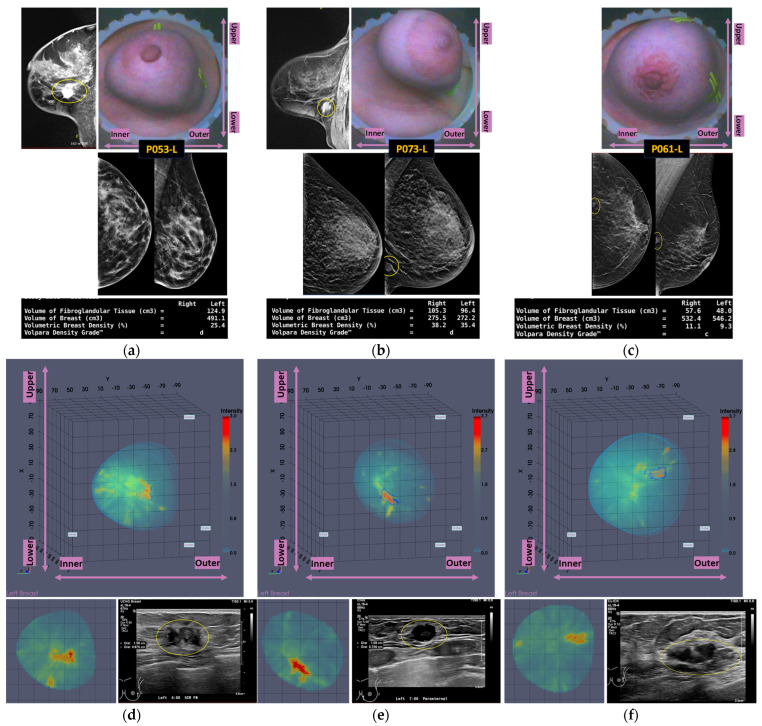
Reference and Wavelia MWBI images for the 3 confusing cases in the malignant-to-benign lesion separability analysis space: (**a**,**d**) P053-L, (**b**,**e**) P073-L, and (**c**,**f**) P061-L. Top row: endoscopic camera view of each breast after positioning in the MWBI scanner—depicted view aligned with the orientation of the MWBI 3D image’s Default View; CC and MLO mammographic views of the breast; Volpara Lab—mammographic computational outputs (Volumetric Breast Density (VBD) and Breast Volume); and sagittal slice highlighting the lesion in the breast MRI scan (if available). Bottom Row: Wavelia MWBI 3D image’s Default View, with the extracted ROI delineated and superimposed in blue; coronal XY slice of the MWBI image cut at the centroid of the clinically relevant ROI in the MWBI image; and snapshot of the ultrasound scan of the breast provided as reference data for the same lesion in this study.

An illustrative subset of the Wavelia MWBI imaging outputs, together with the ultrasound reference scan of the lesion, the X-ray mammogram, and the MRI scan, where available, are depicted in Figure 11 for each of the three cases. Endoscopic camera views showing the position and orientation of the three breasts in the MWBI scanner are also shown in Figure 11.

Lower SNR values are systematically reported for all three cases in Table 3, compared to the stable SNR values reported in Table 2 for the four typical cases of good-quality MWBI scans in the previous section. The CNR values are also lower here, more notably for the Invasive Lobular Carcinoma (ILC) case of Patient 053.

The mammographic images of the breasts provided as reference in Figure 11, together with the associated computational outputs of the Volpara Lab, reveal the highly challenging ROI extraction conditions due to very high breast density in the cases of Patients 053 and 073 (Volumetric Density Grade (VDG) score of d for both P053 and P073, associated with VBD = 25.5% and VBD = 35.4%, respectively).

An additional complexity factor, valid in the cases of Patients 073 and 061, is the location of the lesion in close proximity to the chest wall. These are both cases of small/medium-sized breasts well-inserted in the Wavelia MWBI scanner. The zone in proximity to the chest wall, where the lesions are located as per MRI and X-ray mammographic reference data, is expected to be well-illuminated by the Wavelia scanner. However, the MWBI image in this zone is of limited contrast and diagnostic value due to the high dielectric constant and electromagnetic wave propagation losses induced by the pectoral muscle and bones present in this zone [10,11].

The challenging conditions related to the expected low levels of dielectric contrast of the lesions in the aforementioned breast settings may explain, at least partially, the unclear representation of these lesions and their confusion as either malignant or benign when mapped on the Wavelia MWBI feature analysis space. Nevertheless, by comparative visual inspection of the Wavelia MWBI, the ultrasound, and the MRI scans in Figure 11, it is worth noting that the morphology of the lesions on the Wavelia MWBI images is still quite well-correlated with their morphology on the reference radiological images. In the case of Patient 073, the lesion morphology on the Wavelia image appears to better match the MRI reference image, rather than the ultrasound image. Post-surgery histology data were made available as a reference for this case. The size of the invasive tumor in the excised tissue specimen was 16 mm, matching the 17 mm MRI-based lesion size estimate well. The ultrasound scan provided only partial visibility of this IDC, resulting in a severe underestimation of its size (i.e., 11 mm). The Wavelia MWBI-based estimate of the maximal linear dimension of the lesion was 23 mm. The total tumor size was 49 mm, as per the post-surgery histology report.

### 3.4. Phenomenological Qualitative Assessment of MWBI False Positive Rate in Healthy Breasts

The methodology that was described in Section 2.7 was applied for a preliminary assessment of the Wavelia MWBI false positive rate (unspecified findings) in healthy breasts with no suspicious clinically reportable findings. The healthy contralateral breasts of patients for whom the dominant discrete lesion in the main symptomatic breast was detectable with Wavelia MWBI were included in this analysis. All the patients with bilateral reported lesions or other specific findings in the contralateral asymptomatic breast, such as scar tissue due to prior surgery in the breast, were excluded from this sub-analysis.

The analysis included a total of 41 contralateral healthy breasts of symptomatic patients.

Thirteen unspecified ROIs of non-negligible intensity in the contralateral breast, compared to the intensity of the symptomatic lesion ROI for the same patient, were extracted in total, out of the full analysis set. These could not be attributed to any of the four categories of identifiable MWBI imaging artefacts at the actual stage of development, as illustrated in Appendix B.

**Figure 12:**
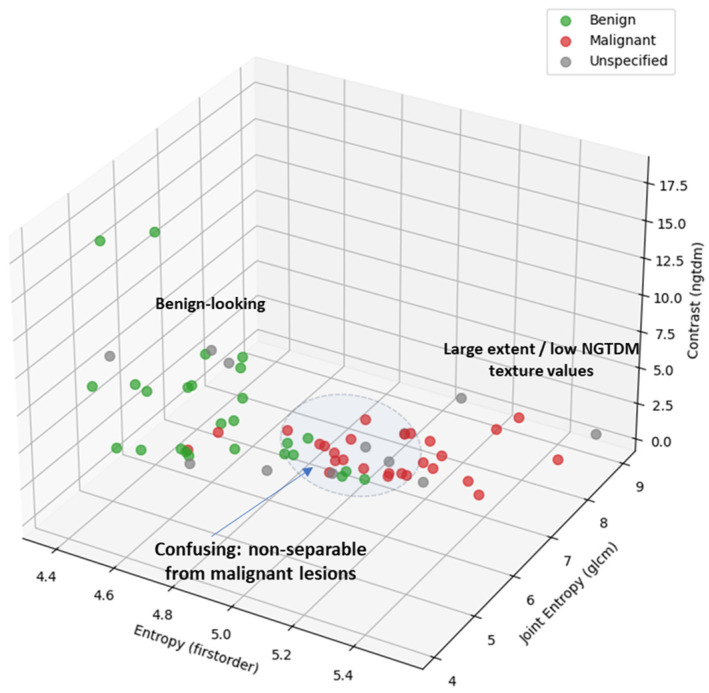
Unspecified findings mapped on the Wavelia MWBI feature analysis space: an indicative 3D feature space depicted, considering three of the features with the highest diagnostic accuracy in terms of discriminating between malignant and benign lesions. An expanded feature space of higher dimension is available for exploitation and analysis.

The unspecified ROIs are represented by points of gray color when mapped on the Wavelia MWBI feature analysis space in Figure 12. It is highlighted on the scatter plot that only 4 out of the 13 unspecified ROIs in a total of 41 healthy breasts appear to be confusing and non-separable from malignant lesions, when analyzed in terms of texture and 1st-order statistics of intensity at this stage of development of the MWBI system.

This phenomenological analysis suggests a ~10% false positive rate for Wavelia MWBI while assuming controlled levels of intensity in the MWBI images.

## 4. Discussion

To our knowledge, Wavelia is the only MWBI prototype system that generates 3D volumetric images of the breast for visual inspection and clinical interpretation, in conjunction with a comprehensive image analysis set per extracted ROI, to support semi-automated detection and characterization of breast lesions based on intensity, shape, and texture features. In this study, the potential of the Wavelia#2 MWBI technology to support discrimination between malignant and benign lesions, further building on the preliminary findings reported for the Wavelia#1 FiH study in [36], is demonstrated in the presented results. In this dataset, more information lies in the texture and intensity statistics–based features. Analysis in a multidimensional radiomic feature space, and not in a restricted three-dimensional space combining a specific and well-defined set of shape and texture descriptors, as optimistically suggested for Wavelia#1 [36], was more appropriate.

A highly diverse clinical study population in terms of breast size, age, breast density, lesion size, and depth of the lesion location in the breast was included in the Wavelia#2 clinical investigation, thus being associated with a set of identified technical challenges for the MWBI system, to handle the geometrical diversity and breast tissue heterogeneity. A multi-modal setting was designed for the MWBI scan data preprocessing scheme towards the standardization of the imaging outputs and controlled specificity, currently a main barrier for clinical validation and acceptance of the Microwave Breast Imaging modality. At this stage of development of the Wavelia MWBI technology, the attribution of the best-fitting mode of operation to each breast scan dataset was performed by the R&D engineering and data analysis team. The process is not fully automated yet for a stand-alone deployment of the system at the clinical investigation site. In future developments, supervised machine learning tools could be employed to train the mode selection process on much larger datasets and ultimately automate it once the critical physical parameters mastering the biology-based interactions of the breast imaging scene with the microwave scanner, overall topology, frequency, etc., have been fundamentally understood and modeled. The implemented multi-modal setting (including three modes) and the phenomenological analysis of the mode selection per category of breast imaging setting, as presented in this study, represent significant progress and a step ahead towards the rationalization of the imaging outputs, modeling of the fundamental underlying physical mechanisms, and ultimate integration with advanced AI tools supporting clinical diagnosis. Automation of the multi-modal imaging setting and the patient positioning guidance will drastically contribute to both the reproducibility and efficiency of the workflow towards the clinical adoption of the MWBI technology.

The challenging conditions related to the expected low levels of dielectric contrast of the lesions in cases of very dense breasts or challenging locations of the lesions in the breast (i.e., close to the chest wall or in the retro-areolar zone) may explain, at least partially, the unclear representation of these lesions and their confusion as either malignant or benign when mapped on the Wavelia MWBI feature analysis space. A good correlation of the morphology of the lesions on the Wavelia MWBI images with the lesions’ morphology on ultrasound and/or MRI reference radiological images was illustrated in the results. A meaningful estimate of the sizes of the lesions was also reported in cases with post-surgery histology reference data available for the tumor size.

In this study, a preliminary understanding and classification of recurrent MWBI imaging artefacts with rationalized and sufficiently identifiable patterns, was addressed and published for the first time.

Following the separation of the recurrent and understood imaging artefacts, a preliminary sub-analysis of the MWBI specificity in asymptomatic contralateral breasts reported only 4 out of the 41 analyzed healthy breasts, with unspecified findings of significant intensity and imaging artefacts that are non-characterizable and not separable from malignant lesions. At the actual Technology Readiness Level of Wavelia MWBI, the intensity level to define an outstanding ROI for clinical reporting and analysis was benchmarked by the symptomatic dominant discrete lesion-related ROI of each patient’s MWBI bilateral breast scan. The following steps will need to be undertaken and achieved to move forward to a more objective quantitative assessment of the MWBI specificity while adhering to the principles of the methodology defined in this study: (a) the recurrent imaging artefacts should be concretely understood and characterized, such that automated detection by means of pattern recognition techniques is enabled, (b) the definition of the significant intensity level for an extractable ROI, should be automatically defined per patient profile, based on models pre-trained on large MWBI scan datasets, generated with upgraded MWBI system prototypes having achieved the generation of standardized imaging outputs, and (c) the confusion (or not) of the residual unspecified findings as malignant lesions should be quantitatively defined by estimating the probability of malignancy for each ROI based on classifier(s) trained in the multidimensional feature analysis space. Once a sufficient maturity level of the MWBI technology is achieved to allow the proper implementation of the aforementioned steps, quantitative validation, including specificity comparisons against established imaging modalities, will be ultimately integrated in future studies.

The Wavelia MWBI imaging procedure is lengthy and will require significant acceleration before clinical acceptance. The impact of the lengthy process for this study was that the MWBI scan of all the biopsy-proven lesions was performed post-biopsy, at least 2 weeks after biopsy, to allow for sufficient healing of the breast. The majority of the MWBI scans being analyzed included a biopsy clip marking the location of the pre-diagnosed lesion in the breast, as per standard-of-care assessments at the University Hospital of Galway. The metallic biopsy clip represents a potential cofounder, especially for lesion characterization, which will need separate attention and analysis in future developments before clinical validation of our findings at a larger and more impactful scale. Future studies should include a greater proportion of clip-free lesions to better assess performance in unmarked tissue.

The electromagnetic waves are more heavily attenuated while propagating through denser breasts. In addition, the dielectric constant is also higher in the case of denser breast tissues, resulting in lower expected levels of dielectric contrast of the malignant tissue against normal breast parenchyma in denser breasts. Both factors (the higher propagation losses attenuating the microwaves and the lower levels of effective dielectric contrast between malignant and background healthy tissue in dense breasts) physically explain the lower SNR and CNR values reported for the test cases presented in Section 3.3, compared to the cases included in Section 3.2. Even though outperforming X-ray mammography in the case of dense and extremely dense breasts shows potential for the MWBI technology based on the available early-phase clinical findings, the dense breasts physically represent more challenging imaging conditions compared to good-quality scans of lower-density breasts, which remain to be further investigated and better quantified in future larger studies. Nevertheless, other factors, such as a low quality of achieved breast positioning, presence of skin folds, and more severe deformability of the breast in the case of fatty breasts in elderly patients, may also compromise overall achieved performances at the current stage of development of the Wavelia MWBI technology.

The overall field of view (FOV) and inclusion of the axillary part of the breast in the MWBI scan are currently limited by the size of the Wavelia probes and the actual configuration of the antenna array. As stated earlier, the uppermost scanning position of the probe array in the Wavelia#2 prototype lies 24 mm below the horizontal level of the examination table of the scanner. In the case of small breasts deeply inserted in the scanner, the upper 20 mm of the scanning zone is typically dominated by the patient’s chest wall. The full pendulous breast can be properly scanned, and the breast tail might even be well-inserted in the scanner, even though challenges associated with the presence of muscles and bones with a high dielectric constant in close vicinity prevent good-quality images from being generated in that zone in the current implementation. In the case of larger breasts, a partial scan of the full breast and no accessibility to the breast tail is effective in the actual setting. Modifications in the circular antenna array configuration to allow better illumination of the imaging scene in closer vicinity to the examination table, as well as a physically relevant modeling of the imaging scene around the border of the posterior pendulous breast, the breast tail, the pectoral muscle, and the chest wall, may allow MWBI images of good diagnostic quality to be generated in that zone. This represents future work beyond the current implementation of the Wavelia scanner.

## 5. Conclusions

Some of the most important findings of the Wavelia#2 prototype testing on human symptomatic breasts are presented in this article for the first time. The potential of the Wavelia#2 MWBI technology in a larger and more diverse dataset compared to the one of the First-In-Human study with the Wavelia#1 prototype, mainly in terms of discrimination between malignant and benign lesions and overall specificity, is demonstrated.

This study focuses on the critical technological evolutions and remaining challenges to handle the diversity of breast geometry and consistency, lesion location in the breast, and lesion histological type before achieving standardization, objective interpretability of the MWBI images, full automation of the imaging suite with reliable outputs, and ultimate clinical acceptance of this new breast imaging modality in the diagnostic setting.

## Figures and Tables

**Table 1:** Malignant-to-benign lesion separability analysis, applying ANOVA on a set of 25 features computed with the PyRadiomics library, and 59 clinically relevant ROIs extracted from the Wavelia MWBI 3D breast images and included in the analysis.

Label Category 1	Malignant		
Label Category 2	Benign		
Metric	ANOVA		
Fixed Bin Number	64		
Use Equal Quantization	No		
Feature Library	Pyradiomics		
Feature Category	Feature Name	Separability Score	*p*-value
Shape	Solidity	3.71	0.059
Shape	Sphericity	7.79	0.007
Shape	Elongation	0.02	0.896
Shape	Flatness	4.85	0.032
First-order	Mean	0.07	0.786
First-order	Median	0.13	0.719
First-order	Variance	0.06	0.809
First-order	Entropy	42.29	0
First-order	Skewness	4.45	0.039
First-order	Kurtosis	3.4	0.07
First-order	Uniformity	23.84	0
GLCM	Autocorrelation	4.83	0.032
GLCM	Contrast	6.94	0.011
GLCM	Correlation	4.95	0.03
GLCM	Difference Average	8.29	0.006
GLCM	Inverse Difference	9.74	0.003
GLCM	Joint Average	6.71	0.012
GLCM	Joint Energy	45.82	0
GLCM	Joint Entropy	55.02	0
GLCM	Sum of Squares	5.37	0.024
NGTDM	Coarseness	33.84	0
NGTDM	Contrast	30.34	0
NGTDM	Busyness	9.08	0.004
NGTDM	Complexity	8.26	0.006
NGTDM	Strength	41.12	0

**Table 2:** Characteristics of typical good-quality ROIs of malignant and benign lesions, extracted from Wavelia MWBI 3D images: columns 1–3 contain reference data, and columns 4–13 are part of the Wavelia MWBI image analysis outputs, with data structured per extracted ROI.

Breast_ID	Histological Label	Cancer Molecular Subtype	Image Quality Metrics		ROI Size [mm]	ROI Shape		Intensity 1st Order Stat		Texture GLCM	Texture NGTDM	
			SNR	CNR	Max Linear Dimension	Elongation	Solidity	Median	Entropy	Joint Entropy	Contrast	Strength
W2P1-01-003_MBI_P001_L001	Malignant (IDC)	Luminal A	9.110	6.390	29.400	0.604	0.664	3.219	5.521	7.979	1.443	32.353
W2P1-01-031_MBI_P001_L001	Malignant (IDC)	Luminal B (HER2-)	9.080	5.500	28.400	0.362	0.665	2.476	5.261	6.447	3.500	42.680
W2P1-01-001_MBI_P001_L001	Cyst		9.190	5.760	15.200	0.454	0.794	2.633	4.934	5.515	5.148	55.562
W2P1-01-023_MBI_P001_L001	Solid Benign		8.710	5.880	15.600	0.467	0.607	3.021	4.703	4.807	9.294	66.092

**Table 3:** Characteristics of confusing ROIs of malignant and benign lesions, as extracted from the Wavelia MWBI 3D images: columns 1–3 contain reference data, and columns 4–13 are part of the Wavelia MWBI image analysis outputs, with data structured per extracted ROI.

Breast_ID	Histological Label	Cancer Molecular Subtype	Image Quality Metrics		ROI Size [mm]	ROI Shape		Intensity First-Oder Stat’s		Texture GLCM	Texture NGTDM	
			SNR	CNR	Max Linear Dimension	Elongation	Solidity	Median	Entropy	JointEntropy	Contrast	Strength
W2P1-01-053_MBI_P001_L001	Malignant (ILC)	Luminal A	7.440	4.520	15.600	0.890	0.592	2.488	5.223	5.849	3.852	48.901
W2P1-01-073_MBI_P001_L001	Malignant (IDC)		8.550	5.820	23.000	0.360	0.600	3.092	4.967	5.279	6.848	47.402
W2P1-01-061_MBI_P001_L001	Solid Benign		8.410	5.470	23.300	0.542	0.694	2.806	5.100	5.647	3.582	40.197

**Figure A1:**
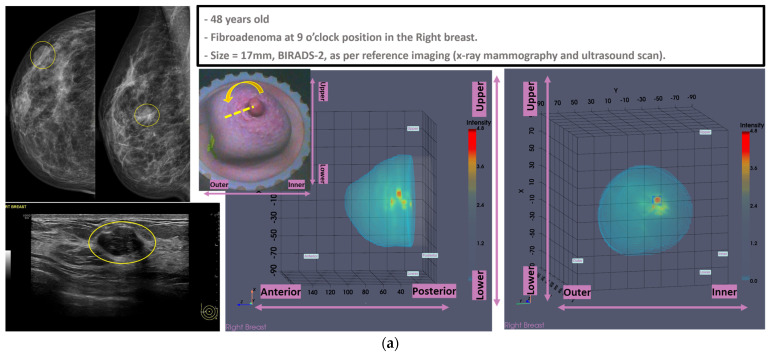

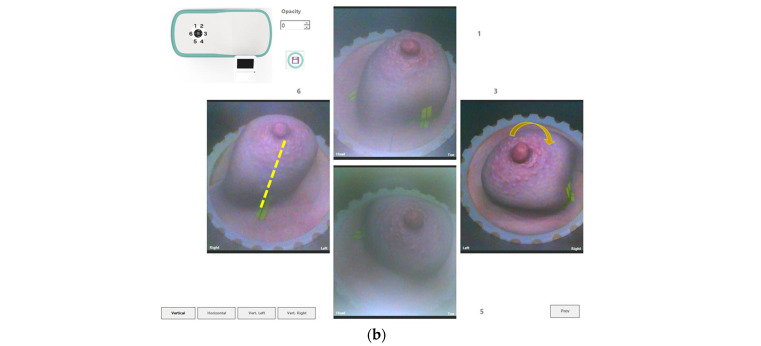
(**a**) P041-R: example of significant mis-location of fibroadenoma in the MWBI 3D images when compared to the X-ray mammography and ultrasound reference images. (**b**) Inspection of the breast position in the scanner, as validated by the operator for the MWBI scan. Views from 4 endoscopic cameras. Significant misalignment of the breast with the scanner XYZ coordinate system. Notable non-verticality of insertion of the pendulous breast in the scanner, potentially explaining the lesion’s location in the Wavelia image.

**Figure A2:**
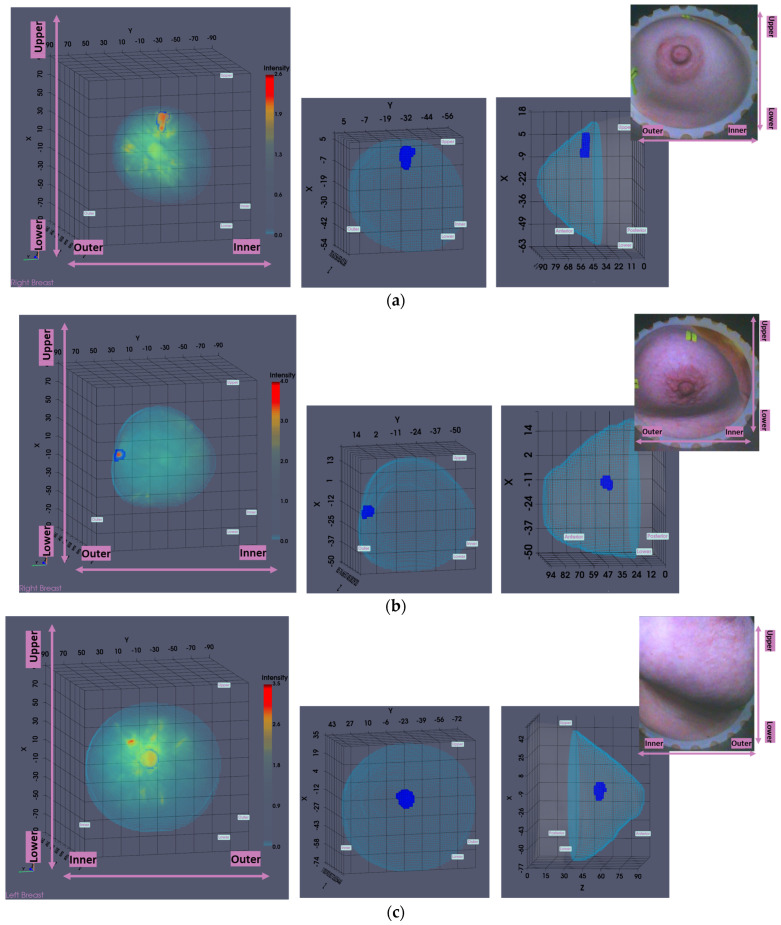

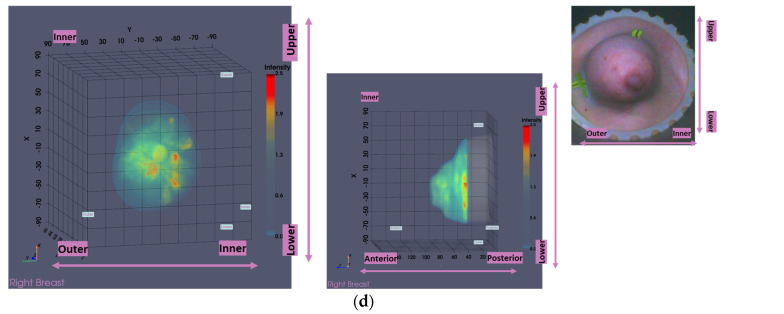
(**a**) P031-R: Tubular-type artefact. (**b**) P061-R: Skin residual artefact. (**c**) P039-L: ‘Circular pattern’ artefact. (**d**) P030-R: Residual of chest within the breast scan.

## Data Availability

The data are not publicly available due to institutional (University Hospital of Galway, Ireland) regulations and non-violation of patients’ privacy.

## Appendix A

### Tolerance for Association of Wavelia MWBI-Based ROIs with the Clinical and Radiological Reference Lesion Location in the Breast

In the Wavelia#2 clinical investigation, there was a set of patient study cases with detected ROIs in the MWBI 3D images that were notably mislocated versus the theoretically expected lesion location in the breast (as per reference radiological data). The availability of the endoscopic camera views of the breast in the Wavelia MWBI scanner was critically important to support the correlation of the Wavelia MWBI ROIs with the reference data in most of these cases. Considerably twisted positioning of the breast in the Wavelia MWBI scanner rendered the association less straightforward in several other cases. Such an ambiguous case is depicted in Figure A1.

Better control of the breast positioning in the MWBI scanner will be required in future MWBI developments towards standardization, quality assurance for the breast scan, and objective clinical association and validation of the MWBI imaging results with reference to the conventional multi-modality imaging data being available at the clinical investigation site.

## Appendix B

### Wavelia#2 MWBI Scanner: Recurrent Imaging Artefacts of Identifiable Pattern

Four categories of imaging artefacts have been defined during the analysis of the Wavelia#2 MWBI clinical investigation dataset.

- Tubular-type artefact: related to slight movement by the patient during the scan. Recognized based on elongation and its proximity to the breast skin contour.
- Skin residual artefact: recognized based on its proximity to the breast skin contour.
- ‘Circular pattern’ artefact: related to residual (unfiltered) antenna-to-antenna direct coupling. Recognized based on its proximity to the center of the scanning zone and its circular shape on the x–y/coronal plane. The circularity of the pattern is directly associated with the circular symmetry of the probe array.
- Residual of chest within the breast scan: artefact recognized based on its proximity to the examination table or its proximity to the border of the selected portion of the scan for imaging in case of the partial scan mode being activated.

An illustrative example for each type of imaging artefact is provided in Figure A2.
